# Chronic irradiation of human cells reduces histone levels and deregulates gene expression

**DOI:** 10.1038/s41598-020-59163-4

**Published:** 2020-02-10

**Authors:** Donna J. Lowe, Mareike Herzog, Thorsten Mosler, Howard Cohen, Sarah Felton, Petra Beli, Ken Raj, Yaron Galanty, Stephen P. Jackson

**Affiliations:** 1 Radiation Effects Department, Centre for Radiation, Chemical and Environmental Hazards, Public Health England, Chilton, Didcot, Oxfordshire OX11 0RQ UK; 2 Wellcome/Cancer Research UK Gurdon Institute and Department of Biochemistry, University of Cambridge, Cambridge, CB2 1QN UK; 3 Institute of Molecular Biology (IMB), 55128 Mainz, Germany; 4 Elizabeth House Surgery, Warlingham, Surrey CR6 9LF UK; 5 Department of Dermatology, Churchill Hospital, Oxford, OX3 7LJ UK

**Keywords:** DNA, Senescence, Epigenetics, Chromatin, Transcriptomics

## Abstract

Over the past decades, there have been huge advances in understanding cellular responses to ionising radiation (IR) and DNA damage. These studies, however, were mostly executed with cell lines and mice using single or multiple acute doses of radiation. Hence, relatively little is known about how continuous exposure to low dose ionising radiation affects normal cells and organisms, even though our cells are constantly exposed to low levels of radiation. We addressed this issue by examining the consequences of exposing human primary cells to continuous ionising γ-radiation delivered at 6–20 mGy/h. Although these dose rates are estimated to inflict fewer than a single DNA double-strand break (DSB) per hour per cell, they still caused dose-dependent reductions in cell proliferation and increased cellular senescence. We concomitantly observed histone protein levels to reduce by up to 40%, which in contrast to previous observations, was not mainly due to protein degradation but instead correlated with reduced histone gene expression. Histone reductions were accompanied by enlarged nuclear size paralleled by an increase in global transcription, including that of pro-inflammatory genes. Thus, chronic irradiation, even at low dose-rates, can induce cell senescence and alter gene expression via a hitherto uncharacterised epigenetic route. These features of chronic radiation represent a new aspect of radiation biology.

## Introduction

The detrimental effects of ionising radiation (IR) on health were observed within a few years of the discovery of X-rays. The link between IR exposure and eventual development of cancer nurtured much research into the mechanisms by which this occurs. Experiments carried out mainly with mice and cell lines uncovered the ability of IR to damage DNA, suggesting that occasional mistakes introduced during DNA repair result in mutations that can drive cells into carcinogenic paths. Several lines of evidence suggest that radiation may also impose biological effects through non-mutational routes, such as the association between radiation and pathologies that do not appear to be initiated or promoted by accumulation of mutations, such as cardiovascular disease^1–4^ and cataract formation^5^, possibly via damage and changes to cellular structures and proteins. Unfortunately, the pace of research in this area has been modest, in part due to the lack of an apparent non-mutational mechanism that could explain the impacts of radiation on these non-cancer diseases.

Another area that remains largely unexplored is the biological consequences of continuous exposure to low doses of radiation. The majority of this comes from natural sources such as radon gas, terrestrial radiation and cosmic rays that penetrate the Earth’s atmosphere. An average person will receive approximately 2.4 mGy per year^6^, yet this varies over 10-fold in different geographical locations worldwide^7^. Although the health impacts of chronic radiation have been questioned from the early days of radiation research, relatively few studies have been carried out. Whether continuous low-level IR would generate proportionally low levels of DNA damage, and whether the repair of these small numbers of lesions incurs proportionally low rates of mutagenesis, are questions that remain to be fully addressed. While it may appear reasonable that risk would have a linear relationship with radiation dose, this has proven to be contentious because experiments have produced equivocal evidence. Various studies suggest that low doses of radiation pose lower^8,9^, predicted^10,11^ or higher^12,13^ risk than expected based on the widely assumed model that there is a linear relationship between dose and risk. It is impossible to ascertain which of these is correct because the number of reported studies is low, and they come from a wide range of experimental sources, which do not lend themselves to direct comparison. Furthermore, conceptual extrapolation of our understanding of DNA damage signalling induced by high and acute doses of radiation may be incorrect, as DNA damage inflicted by low-dose chronic radiation is very different. It is unclear whether there is a threshold of DNA damage that must be breached for cells to respond, and whether continuous and consecutive DNA damage will be tolerated or ignored by the cell. As it has been suggested that persistent DNA-damage signalling can drive cells into senescence^14^, it may be that repeated, albeit low levels of, DNA damage from chronic radiation prevents complete diminution of DNA damage signals below a theoretical minimum level or interval, thereby causing cells to initiate and progress into a senescent state. There is increasing appreciation of the importance of these issues because of the growing use of medical procedures such as computed tomography (CT), which can give more than an average year’s total radiation dose in a single scan^15^. However, the experimental model, endpoint and risk measure will always be critical to this assessment.

Outside the field of radiobiology, attention is increasingly drawn to the importance of non-genetic changes in cancer and non-cancer pathologies. These changes include those mediated by modifications of DNA (such as methylation), as well as modifications of histones at multiple positions (including phosphorylation, methylation and acetylation) and other DNA-binding proteins, and changes in expression of non-coding RNAs; all of which can regulate chromatin state and gene expression^16,17^. Thus, radiation can contribute to pathologies that are not dependent on mutations, through epigenetic alterations. Indeed, we have previously demonstrated that the *CD44* gene promoter is de-methylated following irradiation, resulting in over-expression and presentation of this highly adhesive protein on the apical membrane of endothelial cells, increasing the risk of atherosclerotic plaque development^18^. Indeed, it is now clear that radiation can lead to histone modifications^19–21^, a key paradigm for which being the well-documented effect of IR on the H2A histone variant, H2AX (H2AFX). When DNA is damaged by radiation or alternative causes to yield double-strand breaks (DSBs), H2AX becomes phosphorylated on its C-terminal tail at serine 139 and is known as γH2AX^22,23^. This occurs at and on the chromatin flanking the DSB sites^20^. H2AX phosphorylation in response to DSBs is carried out by the protein kinase ATM in addition to other phosphatidylinositol 3-kinase like kinases ATR and DNA-PK^24^. γH2AX then triggers the recruitment of other cellular proteins to mediate a DNA damage response (DDR), which involves activation of DNA repair mechanisms as well as intracellular signalling processes that impact on various aspects of cell physiology and can lead to temporary cell cycle slowing or arrest, long term cell cycle arrest and/or cell senescence or programmed cell death. The key roles of this histone mark are demonstrated by reduced survival of *H2AX−/−* mice following whole body irradiation^25^ and increased chromosomal aberrations in their embryonic stem cells^26^. Other histone-related responses to radiation include ubiquitylation of histones H2A and H2B^27,28^, several modifications on histones H3 and H4^19^, localised removal of H2A.Z (H2AFZ)^29–32^, augmented levels of histone H2A.J (H2AFJ) and subsequent epigenetic augmentation of inflammatory gene expression^33^.

Here, we describe studies in which we have continuously exposed primary cells isolated from human skin to ionising γ-radiation. We report that radiation exposures estimated to inflict fewer than one DSB per hour per cell, decreased cell proliferation and increased cellular senescence. Moreover, we document that these senescent cell populations display a reduction in histone levels and a concomitant increase in nuclear size and global gene expression. We show that these changes are associated with pronounced changes in gene expression, causing alterations in protein expression, some of which are pro-inflammatory and reflect an aged-cell phenotype. We discuss how the long-term presence of senescent cells harbouring these features in irradiated tissues might comprise a pathological route by which IR imposes its effects on health.

## Materials and Methods

### Isolation, culture and treatment of primary cells

Primary cells were isolated from human neonatal foreskin removed for routine circumcision, or adult facial skin following minor dermatology procedures. Tissue was transported the same day and digested overnight at 4 °C with 0.5 mg/ml liberase DH (Roche, 5401089001) in Epidermal Keratinocyte Medium (CellnTech, CnT-07). The following day, the epidermis was removed using sterile instruments and then pressed in trypsin-EDTA to form a single cell suspension, pelleted and resuspended in keratinocyte medium (CnT-07). Cells were seeded on collagen/fibronectin coated flasks for keratinocyte isolation. To isolate fibroblasts, dermal pieces were grown in DMEM supplemented with 10% FBS (Gibco) and grown as explants. Remaining dermal tissue was digested in 2.5 mg/ml collagenase in HBSS (with added calcium and magnesium) at 37 °C with frequent agitation for 1 h, passed through a 70 μm cell strainer and selected using CD31 magnetic Dynabead positive selection (Life Technologies, 11155D) and seeded on a gelatin-coated flask in Endothelial Cell Growth Medium MV (PromoCell, C-22020). Transport and initial isolation were carried out with double concentration of antibiotics, followed by 7 days in normal concentration (100 U penicillin, 0.1 mg streptomycin, 10 µg gentamycin and 0.25 μg amphotericin B per ml); routine culture and experiments were carried out without antibiotics. Cells were passed by washing with HBSS and trypsinisation, followed by soybean trypsin inhibitor and resuspension in the appropriate medium and seeded at 1:3–1:5 ratio with coating used for initial isolation.

The human cell line RPE-1 (SNP6 verified, from Professor J. Pines - The Institute of Cancer Research, London) was used to generate CRISPR-Cas9 induced clonal knock-outs of p53 and ATM as described previously^34,35^. Cells were grown in DMEM/F12 Ham (Sigma, D6421) supplemented with 10% FBS, pen/strep, glutamine and sodium bicarbonate. RPE-1 knockout cultures were exposed to chronic IR alongside puromycin resistance knockout (wild type) controls.

### Exposure to IR

Cells were chronically irradiated by exposure to a gamma-emitting Cs-137 source in a custom-built irradiator (Gemini Technologies) at 37 °C, 5% CO_2_, high humidity and under constant exposure to low levels of IR except when removed for routine media change. Unirradiated controls were cultured in identical culture conditions without IR. Dose rates of 6–20 mGy/h were achieved by using lead filters and variable distances from the source. Unless otherwise stated, cells were chronically irradiated for 7 days. For acute irradiation, cells were exposed to X-rays in an AGO X-ray System (CP/1601) with 250 kV, 13 mA and 60 cm from the source, giving a dose rate of 0.5 Gy/min.

### Assays for cellular responses to IR

Apoptosis was quantified using Caspase-Glo 3/7 luminescence assay (Promega, G8091). Senescence staining was carried out using SA-β-Galactosidase Staining Kit (Cell Signalling Technologies, 9680) and quantified using Galacto-Light Plus Beta-Galactosidase Reporter Gene Assay System (Invitrogen, T1007) following the manufacturer’s protocol, except modified to use lysosomal reaction buffer (100 mM sodium phosphate pH 6, 20 μM MgCl_2_).

### Indirect immunofluorescence analyses

Cells grown on collagen/fibronectin coated coverslips were fixed with formalin for 15 minutes, permeabilised with 0.1% Triton X-100, blocked with 2% FBS/HBSS and incubated with primary antibody for 1 h followed by washing and Alexa-conjugated secondary incubation, further washes, nuclear staining with 1 ug/ml DAPI and mounting. See Table 1 for list of antibodies used.

**Table 1:** List of antibodies used.

	Supplier	Cat. No.	Application	Dilution
53BP1	Millipore	MAB3802	IF	1:200
ATM	Abcam	ab32420	WB	1:5000
ATM-P (S1981)	Abcam	ab81292	WB	1:5000
Chk1-P (S345)	Cell Signalling	2348	WB	1:1000
GAPDH	Santa Cruz	sc-25778	WB	1:10000
H2A.J	Active Motif	61793	WB	1:1000
H2AX	Bethyl Lab	A300-082A	WB	1:5000
γH2AX	Cell Signalling	9718S	IF/WB	1:200/1:5000
Histone H1	Santa Cruz	sc-8030	WB	1:500
Histone H2A	Abcam	ab18255	WB	1:2000
Histone H2A.Z	Abcam	ab150402	WB	1:1000
Histone H2B	Abcam	ab1790	WB	1:20000
Histone H3	Novus Biologicals	NB500-171	WB	1:20000
Histone H4	Novus Biologicals	NBP2-16848	WB	1:10000
Lamin B1	Abcam	ab16048	WB	1:5000
p21	Abcam	ab18209	WB	1:5000
p53	Santa Cruz	sc-126	WB	1:10000
p53-P (S6)	Cell Signalling	9285	WB	1:1000
p53-P (S15)	Cell Signalling	9284	WB	1:1000
p53-P (S46)	Cell Signalling	2521	WB	1:1000

IF = Immunofluorescence; WB = Western Blot.

### Quantification of DSBs

Following standard immunofluorescence staining of 53BP1, discreet foci (used as a surrogate for DSBs) were quantified per cell using fluorescence microscopy. Samples were scored blind in a minimum of 200 cells per radiation condition.

### Cellular drug treatments

To investigate protein degradation, proteasome was inhibited with 5 μM MG132 (Sigma, M7449) or lysosomal autophagy inhibited with 10 μM chloroquine (Sigma, C6628) for 24 h. For kinase inhibition experiments, ATM was inhibited with 10 μM Ku55933 (Selleck, S1092) and ATR was inhibited with 1–10 uM VE-821 (Selleck, S8007) for the duration indicated. Apoptosis was induced with 1 μM staurosporine (Enzo Life Sciences, ALX-380-014) for 4–6 hours. ROS was inhibited using 2 mM N-acetyl-L-cysteine (NAC) for the duration of the experiment (7 days).

### Protein analysis by western blotting

Cells were harvested by washing cell monolayers twice with cold HBSS, scraped into a 1.5 ml tube, centrifuged and the pellet resuspended in HBSS containing Halt phosphatase and protease inhibitors (Thermo Scientific, 78429) then lysed with 1% SDS in 50 mM Tris pH 8.0. DNA was fragmented using Qiashredder (Qiagen, 79656) and protein concentration quantified using BCA assay (Thermo Scientific Pierce, 10056623 and 10475944) and measuring absorbance at 562 nm. Equal protein amounts were run on a polyacrylamide gel at 100 V for 1.5–2 h before transferring to PVDF membrane at 2.5 A, 25 V for 7 minutes using TransBlot Turbo (Bio-Rad). Membranes were blocked in 5% non-fat milk in TBS-T then incubated overnight with primary antibodies (Table 1), washed three times with TBS-T, incubated for 1 h with HRP conjugated secondary antibody washed three times with TBS-T and exposed to X-ray film after ECL incubation (Immobilon Western HRP Substrate Reagent/Peroxide Substrate, Millipore, WBKLS0500). All antibody incubations were carried out in 5% non-fat milk in TBS-T.

### MS sample preparation

For SILAC (stable isotope labelling of amino acids in cell culture) experiments, primary fibroblasts were grown in heavy (R10K8), light (R6K4) or unlabelled SILAC DMEM (Dundee Cell Products) for 8–10 population doublings then exposed to 0, 6 or 20 mGy/h for 7 days. Cell pellets were collected, lysed with LDS buffer supplemented with 1 mM DTT, heated at 70 degrees for 10 min. Samples were then quantified and pooled in mixed combinations to provide triplicate samples. Proteins were alkylated by the addition of 5.5 mM chloroacetamide for 30 min and resolved on 4–12% gradient SDS-PAGE gels (NuPAGE Bis-Tris Precast Gels, Life Technologies). The gels were stained using the Colloidal Blue Staining Kit (Life Technologies) and proteins were digested in-gel using trypsin. Peptides were extracted from gel and desalted on reversed-phase C18 StageTips^36^.

### MS analysis

Peptide fractions were analysed on a quadrupole Orbitrap mass spectrometer (Q Exactive Plus, Thermo Scientific) equipped with a UHPLC system (EASY-nLC 1000, Thermo Scientific)^37^. Peptide samples were loaded onto C18 reversed phase columns (15 cm length, 75 μm inner diameter, 1.9 μm bead size) and eluted with a linear gradient from 5 to 30% acetonitrile containing 0.1% formic acid in 3 h. The mass spectrometer was operated in data dependent mode, automatically switching between MS and MS2 acquisition. Survey full-scanMS spectra (m/z 300–1700) were acquired in the Orbitrap. The ten most intense ions were sequentially isolated and fragmented by higher energy C-trap dissociation (HCD)^38^. Peptides with unassigned charge states, as well as with charge states less than +2 were excluded from fragmentation. Fragment spectra were acquired in the Orbitrap mass analyser.

### Peptide identification

Raw data files were analyzed using MaxQuant (development version 1.5.2.8)^39^. Parent ion and MS2 spectra were searched against a database containing 92,578 human protein sequences obtained from the UniProtKB released in December 2016 using Andromeda search engine^40^. Spectra were searched with a mass tolerance of 6 ppm in MS mode, 20 ppm in HCD MS2 mode, strict trypsin specificity and allowing up to two missed-cleavages. Cysteine carbamidomethylation was searched as a fixed modification, whereas protein N-terminal acetylation and methionine oxidation were searched as variable modifications. The dataset was filtered based on posterior error probability to arrive at a false discovery rate below 1% estimated using a target-decoy approach^41^.

### RNA-seq differential gene expression

RNA was extracted (Direct-zol, Zymo). Quality and concentration were tested using (RNA BR assay kit, Quabit) both following the manufacturer’s protocol. 50 ug of RNA was used for whole transcriptome Illumina Hi-seq. 4000_150PE sequencing. Paired-end sequencing reads were aligned to the human genome hg1 using Tophat. Bam files from the same sample sequenced across different lanes were merged using Samtools merge. Features were counted with HTSeq. QC and differential gene expression analysis was carried out.

### qRT-PCR gene expression analysis

RNA was extracted using the RNeasy Mini Kit, QIAshredder and RNase-free DNase set (all Qiagen) according to the manufacturer’s protocol and eluted in 30 μl RNase free water. RNA was quantified using Nanodrop spectrophotometric analysis (Labtech) measuring absorbance at 260 nm; 260/280 and 260/230 ratios were monitored to indicate sample purity. 1000 ng RNA was reverse transcribed to cDNA using High Capacity RT kit (AB Bioscience). In triplicate, 10 μl of PCR reaction mix (1 μl sample cDNA, 5 μl PerfeCTa SYBR Green Supermix (Quanta Biosciences), 0.3 μl of each F and R primers (3.3 μM) and 3.4 μl nuclease free water) was transferred to a 100-well rotor along with ‘no reverse transcription’ and ‘no transcript’ controls, and run on a Rotor Gene Q PCR machine for: 95 °C for 2 minutes, 40 cycles of 95 °C for 10 seconds then 60 °C for 60 seconds, followed by a melt from 65 °C to 95 °C in 1 °C intervals; with optimisation before the first acquisition and channel was set to green. Ct values were normalised to the housekeeping gene HPRT and compared between irradiated and control samples to give an average fold change by calculating 2^−ΔΔCt^.

### Detection of reactive oxygen species

To detect cellular ROS, we used a DCFDA/H2DCFDA ROS Assay Kit (Abcam, ab113851), following manufacturer’s instructions. For both plate reader measurement, 50,000 cells were seeded in triplicate in a 96 well plate 24 hours prior to analysis of fluorescence. For flow cytometry analysis, cells were incubated with the dye during incubation with or without chronic radiation, trypsinised and 1000 low-PI gated cells measured to establish mean fluorescence within the population. Cells were treated with 200 mM tert-butyl hydroperoxide for 1 hour as a positive control.

### Analysis of nuclear area and RNA synthesis

To measure nuclear area, cell monolayers were chronically irradiated as described and when almost confluent stained with 1 μg/ml DAPI (Sigma, D8417). Nuclei from a minimum of 200 cells or 5 fields were automatically detected and mean nuclear area calculated. Global RNA synthesis was measured using 2 h pulse labelling with Alexa 488-labelled EU and detected using RNA Click-IT HCS kit (C10327, Invitrogen) following the manufacturer’s protocol and quantified by automatic detection and intensity measurement using Nikon Eclipse Ti fluorescent microscope and Elements software. Box plots were generated using BoxPlotR (http://shiny.chemgrid.org/boxplotr/).

### Ethical approval and informed consent

Informed consent was obtained prior to collection of human skin samples with approval by the Oxford Research Ethics Committee; reference 10/H0605/1. All experiments were performed in accordance with relevant guidelines and regulations.

## Results

A confounding factor in experimental biology is the use of cell lines, the majority of which harbour known and unknown mutations and/or chromosomal rearrangements, amplifications and aneuploidies. Thus, this limits a researcher’s ability to accurately generalise observations made using them. Another factor that limits such generalisations is the intrinsic differences between cell types and cell lines in regard to how they respond to challenges such as radiation. While it is impossible to test all cell types that constitute the body, we have endeavoured to do so for a limited number of primary, non-transformed cell types in most of the experiments described herein. To this end, we established and optimised the isolation and culture of keratinocytes, fibroblasts and microvascular endothelial cells from neonatal foreskins. While most of the experiments below were carried out with human primary fibroblasts, many of them were also replicated with the other cell types and from multiple donors, thereby greatly reducing the potential for donor-specific influences.

### Low dose rates of chronic radiation inhibit cell proliferation

**Figure 1:**
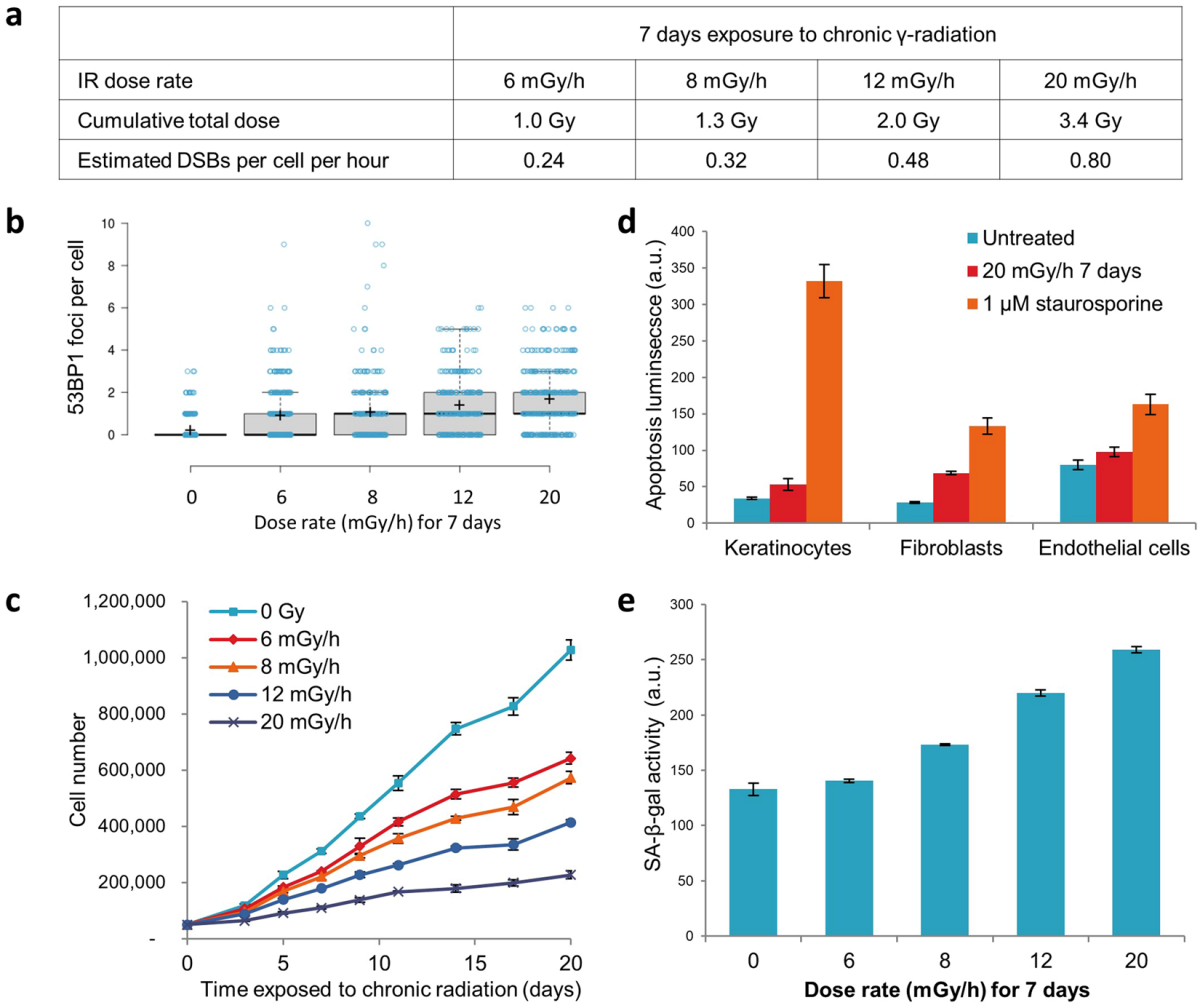
Chronic low dose γ-radiation retards cell growth and induces cell responses to DNA damage. (**a**) Dose rates used for chronic γ-radiation exposures, corresponding cumulative doses over a 7-day period and DSB estimates based on 1 Gy generating 40 DSBs in a human cell (Olive, 1998). (**b**) Quantification of DSBs by immunofluorescence staining of 53BP1 foci in primary fibroblasts exposed to chronic radiation for 7 days (>200 cells measured per condition, displayed as blue circles). Mean numbers of foci per cell are shown by crosses, and Tukey analyses are shown by boxes. (**c**) Proliferation rates of fibroblasts exposed to various dose-rates of chronic γ-radiation, data shown as triplicate measurements of one donor. (**d**) Chronic radiation-induced apoptosis in fibroblasts measured luminescence generated by caspase 3 cleavage in a population of cells as a positive marker of apoptosis. Staurosprorine treated cells were used as positive control. (**e**) Induction of senescence by chronic radiation in primary fibroblasts as measured by GalactoLight assay for senescence-associated beta-galactosidase activity after 7 days of chronic irradiation at the stated dose rates. The graph shows the total activity in a population of cells of the same number, measured in arbitrary units of luminescence (a.u.).

We exposed primary keratinocytes and fibroblasts to chronic γ-radiation at dose rates ranging from 6 mGy/h to 20 mGy/h for 7 days; doses predicted to produce relatively low numbers of DNA double-strand breaks (Fig. 1a). As a case in point, γ-radiation delivered at 20 mGy/hr is estimated to inflict fewer than one DSB per cell per hour. These predictions were in line with our experimental quantification of numbers of TP53-binding protein 1 (53BP1) foci, each of which represents the protein’s accumulation at a DSB site (Fig. 1b). Despite the small numbers of DSBs, these doses of radiation clearly impeded cell proliferation in a dose-dependent manner (Fig. 1c). This likely reflects a combination of cell death, which was indeed somewhat augmented upon irradiation (Fig. 1d), and cellular senescence (as measured by staining for senescence-associated beta-galactosidase activity under acidic conditions; Fig. 1e). To address the likelihood that these effects were mediated by increased level of reactive oxygen species (ROS), we measured ROS levels and found them to be comparable in chronically irradiated and unirradiated control cells (Fig. S1a). This would suggest that the effects above were mediated independently of ROS.

### Core histone protein levels are reduced by chronic radiation exposure

**Figure 2:**
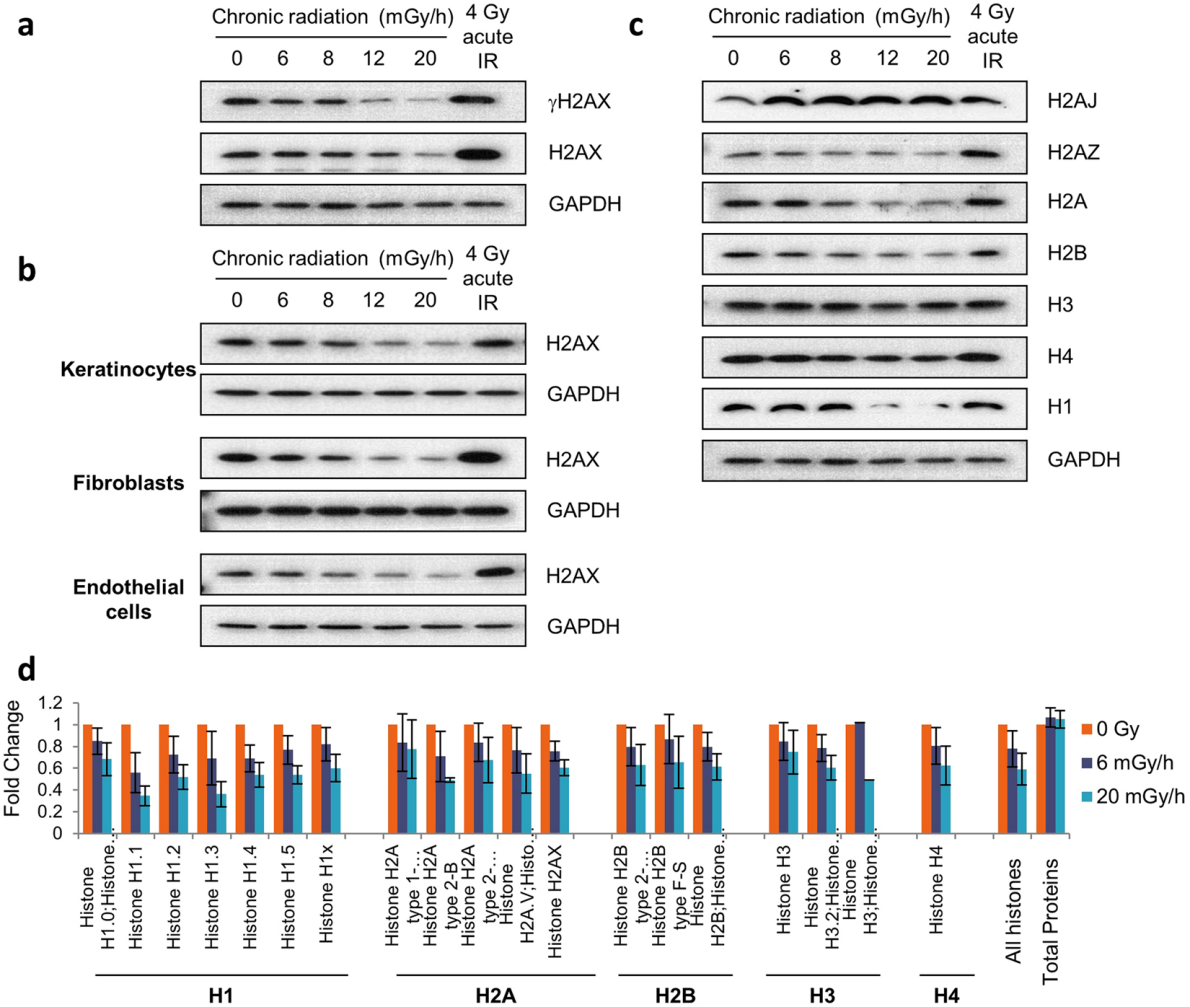
Chronic γ-radiation reduces histone levels. (**a**) Western blot analyses of histone H2AX and γH2AX in primary fibroblasts exposed to various dose-rates of chronic γ-radiation for 7 days. Cells irradiated with a single acute dose of 4 Gy X-ray were included as control. (**b)** Effect of chronic γ-irradiation on H2AX levels in three different isogenic primary cell types from a different donor to that used in (**a**) at the same dose rates as in (**a**). (**c**) Immunoblots of other histones in chronically irradiated primary fibroblasts. (**d**) All significant histone level changes detected by SILAC LC-MS/MS protein analyses of samples from primary fibroblasts exposed or mock-exposed to chronic γ-radiation for 7 days.

While using western immunoblotting to analyse various DDR proteins in un-irradiated and irradiated cells, we observed a counter-intuitive change in H2AX phosphorylation. Perhaps surprisingly, the level of γH2AX, which is well-documented to be induced by DNA-damaging agents such as radiation, showed a dose-dependent decrease in extracts of chronically-irradiated primary fibroblasts (Fig. 2a). Strikingly, analysis of H2AX protein levels revealed that this was also associated with a reduction in the overall levels of this protein following chronic radiation. This reduction in histone levels is even more noteworthy when the amount of DNA in each loaded sample was measured and found to be comparable between samples, with a marginal increase in irradiated ones; making the reduction of histones relative to DNA even more stark (Fig. S2a). To determine whether this phenomenon is limited to fibroblasts or to cells from a specific donor, we isolated primary keratinocytes, fibroblasts and endothelial cells from another donor and exposed these to chronic radiation at similar dose rates. Again, we observed reductions in histone H2AX levels, indicating that the reduction in H2AX was neither cell-type nor donor dependent (Fig. 2b). Importantly, these cells were competent with regards to phosphorylating the H2AX protein, which they did effectively when they were irradiated with 4 Gy of X-ray. Histone H2AX level reduction was also seen with non-skin cells, namely the hTERT-immortalised retinal pigmented epithelium (RPE-1) cell line derived from human eye lens (Fig. S2b). Collectively, these studies suggested that H2AX histone levels are reduced as a generalised outcome of low dose chronic irradiation.

When we used western immunoblotting to assess the levels of other core histone proteins (H2A, H2A.Z, H2B, H3 and H4) and histone H1, we noted that these also displayed general declines in response to chronic irradiation, albeit to differing extents (Fig. 2c). By contrast, we observed that chronic irradiation led to enhanced levels of histone H2A.J, a histone variant recently shown to accumulate specifically in senescent cells^33^. To extend our analyses further, we employed a quantitative protein analysis approach – stable isotope labelling of amino acids in cell culture (SILAC) followed by quantitative mass spectrometry analyses – to measure and compare histone levels in irradiated and non-irradiated cells. The results of this analysis (Fig. 2d) confirmed that normalized to general protein levels, amounts of all core histones were indeed reduced by irradiation in a dose-dependent manner, with histones overall showing, on average, a reduction of 22% with 6 mGy/h and 41% at 20 mGy/h chronic radiation for 7 days (Fig. 2d). Importantly, this contrasted with the absence of change in the levels of total cellular proteins.

Besides highlighting the effects of chronic radiation on histone levels, SILAC analysis also revealed specific reductions in levels of non-histone chromatin-associated proteins such as HMGB and SMC, as well as levels of certain proteins involved in DNA replication and cell cycle progression, including MCM2-7, SMC1A/3, and the DNA polymerase delta catalytic subunit (POLD1) (Fig. S3 and Table 2). Notably, the levels of HMGB2^42,43^, Lamin B1 (LMNB1)^44–46^ and TMPO^47^, which have all been reported to be lower in senescent cells were also reduced in irradiated cells, in keeping with the rise of cellular senescence as indicated by staining for senescence-associated beta-galactosidase activity (Figs. 1e and S4). Consistent with this, increases in levels of p16 (CDKN2A) (1.23 fold) and p21 (CDKN1A) (1.48 fold), which are often augmented in senescent cells^48–50^, were also detected in chronically-irradiated cells, but do not appear in Fig. S3 as the increases were below the arbitrary threshold of 1.5 fold change. Other proteins displaying increased levels upon chronic radiation included structural and metabolic proteins, a selection of DDR proteins, anti-proliferative proteins and components of the ubiquitin proteasome system. Upregulated proteins of particular note were the non-homologous end-joining repair protein XRCC4, RRMB2 that catalyses the synthesis of deoxyribonucleotides required for DNA repair and replication, the ubiquitin ligase complex component FBXO44 and the p53-regulated pro-apoptotic PERP protein (Fig. S3).

**Table 2:** Pathway analysis of SILAC-based proteomic changes.

Pathway ID	Pathway description	Observed gene count	False discovery rate	Proteins in pathway
3030	DNA replication	11	1.31E-12	FEN1, MCM2, MCM3, MCM4, MCM5, MCM6, MCM7, POLD1, RFC2, RFC3, RFC4
4110	Cell cycle	10	1.30E-05	CREBBP, MAD2L1, MCM2, MCM3, MCM4, MCM5, MCM6, MCM7, SMC1A, SMC3
3430	DNA mismatch repair	5	0.000123	MSH6, POLD1, RFC2, RFC3, RFC4
3410	DNA base excision repair	5	0.000607	FEN1, HMGB1, MPG, PARP1, POLD1
3420	DNA nucleotide excision repair	4	0.0366	POLD1, RFC2, RFC3, RFC4

KEGG (Kyoto Encyclopedia of Genes and Genomes) pathway analysis of proteins with >1.5-fold reduction in fibroblasts exposed to 20 mGy/h chronic γ-radiation. Analysis was carried out using STRING (https://string-db.org/v10.5). Note that no significant pathways were identified for upregulated proteins.

### Histone reductions occur in response to factors inducing cell senescence

**Figure 3:**
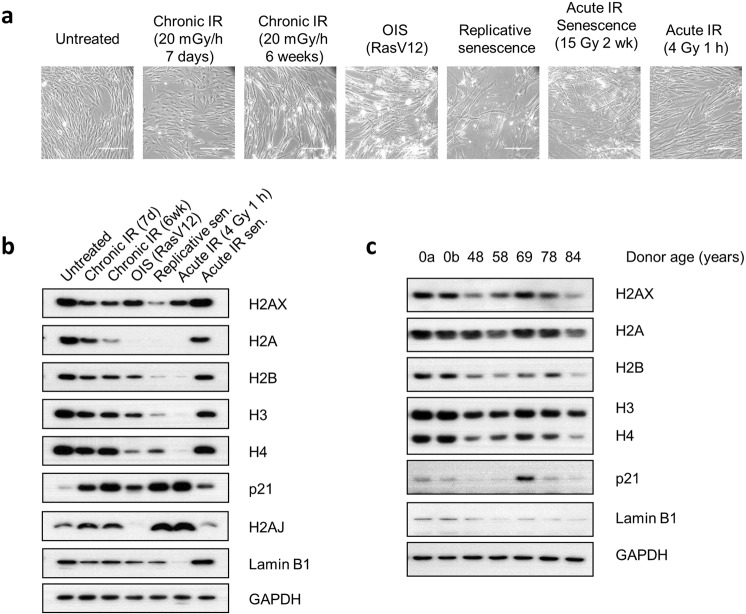
Reduced histone levels in senescent cells is induced *in vitro* by different means, and *in vivo* from aged donors. (**a**) Phase-contrast images of primary fibroblasts induced into senescence by chronic γ-radiation, oncogene over-expression or exhaustive replication (replicative senescence), and DNA damage from a single acute 4 Gy X-ray dose at an early time-point (1 hour) as a control for DNA damage without senescence. Scale bars 200 µm. (**b**) Western immunoblot analyses of histones in fibroblasts described in (**a**). (**c**) Histone levels in dermal fibroblasts isolated from human neonatal (age 0, donors a and b) and adult donors.

If histones were indeed reduced in irradiated cells that became senescent, then it would stand to reason that such reductions would also be expected to accompany cellular senescence triggered by other stimuli. To this end, we compared histone levels of chronically-irradiated cells with those of cells triggered to undergo oncogene-induced senescence (OIS) by Ras overexpression, and cells that had undergone replicative senescence. Senescence was confirmed by enlarged cell morphology (Fig. 3a), increased p21 and H2A.J levels, and decreased lamin B1 (Fig. 3b). These studies showed that the levels of core histones were indeed reduced considerably in all senescent cells regardless of the route by which senescence was induced. Similar findings were also obtained with primary keratinocytes (Fig. S5). These results thus strongly supported a model in which histone reductions in chronically-irradiated cells was associated with these cells having become senescent. An interesting exception to the trend of histone loss was seen with OIS, where H2AX reduction was modest and with no rise in H2A.J (Fig. 3b), suggesting that the senescent state of these cells was different from radiation-induced and replicative senescence.

Since senescent cells are reportedly present in greater numbers in aged human tissues than in tissues from younger individuals^51,52^, we assessed whether the senescence-associated changes in histone levels that we had observed were detectable *in vivo*. To this end, we isolated primary dermal fibroblasts from human donors ranging in age from 48 to 84 years old and compared their histone levels to those of neonatal donors. We found that samples from all older donors, except for one (a 69-year-old individual, who also had a marked increase in p21 levels), had visibly lower amounts of histones than controls (Fig. 3c), suggesting that the loss of histones observed in senescent cells *in vitro* also occurs in general *in vivo* upon increasing age.

### Chronic γ-radiation-induced senescence and histone reduction rely on TP53 but not ATM

**Figure 4:**
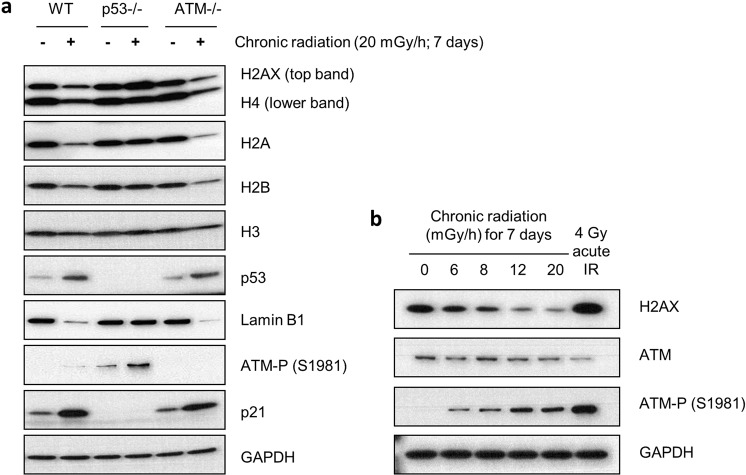
p53 but not ATM is required for chronic γ-radiation-induced histone reductions. (**a**) Western blots of histone proteins in wild-type, *TP53*^*−/−*^ and *ATM*^*−/−*^ RPE-1 cells following exposures to 20 mGy/h chronic radiation for 7 days. (**b**) ATM activation, as shown by auto-phosphorylation on S1981, in response to chronic radiation in primary fibroblasts.

To characterise the signalling mechanisms underpinning our observations, we examined the potential role of the tumour suppressor TP53 (p53) due to its key roles in regulating transcription and senescence in response to DNA damage^53,54^. Thus, we chronically irradiated wild-type, p53 proficient RPE-1 cells alongside derivatives of these cells rendered p53 null by CRISPR-Cas9 genome engineering^34^, at 20 mGy/h for 7 days. As with all other cell types analysed, chronically-irradiated parental RPE-1 cells responded by reducing their levels of all core histone proteins tested in a manner that was accompanied by increased p21 and decreased Lamin B1 expression (Fig. 4a). By contrast, in *TP53*^*−/−*^ cells, histone levels were not reduced upon chronic γ-radiation; and in accord with this, there was no p21 induction, or reduction of Lamin B1 expression in such cells, highlighting the requirement for p53 activity for these responses. p53 is activated by phosphorylation at several sites^55^. We show considerable phosphorylation at serine 15 (Fig. S6a), a site known to be central in DNA-damage response. A small increase in phosphorylation is seen at serine 6 (also reported to be activated by genotoxic stress). However, no phosphorylation was detected at serine 46 or any other sites probed for. Since many p53-regulated responses require its activation by phosphorylation, most notably at serine 15, we investigated the role of ATM, which is the early p53-activating kinase in response to DSBs and required for chronic radiation-induced p53 serine 15 phosphorylation (Fig. S6b). Not surprisingly, as measured by its increased auto-phosphorylation on serine 1981, the ATM protein was itself activated by chronic radiation in both primary fibroblasts and RPE-1 cells (Figs. 4b and S2b). However, when RPE-1 cells lacking ATM were exposed to chronic radiation, histone levels were reduced essentially as efficiently as in the wild-type *ATM*^*+/+*^ setting (Fig. 4a and S2b), showing that ATM is not essential for the observed reductions in histone levels in response to chronic irradiation. This was further supported by experiments using primary fibroblasts exposed to chronic radiation in the presence of the ATM inhibitor KU55933^56^ for 24 h prior to harvesting or for the entire duration of the 7-day exposure (Figs. S2c). While it did at first appear surprising that p53 but not ATM was required for reducing histone levels in response to chronic irradiation, we noted that p53 protein was upregulated, with concomitant increase of its downstream target p21, in chronically irradiated RPE-1 cells independently of ATM status (Fig. 4). While there may be other explanations, these findings suggested that in ATM’s absence, ATR and/or DNA-PK might serve to activate p53 in response to chronic irradiation. Whatever the case, it was clear from the reduced levels of lamin B1 and increased levels of p21 (and changes in cell morphology), that unlike the situation with *TP53*^*−/−*^ cells, *ATM*^*−/−*^ cells were not impeded from becoming senescent in response to chronic irradiation. Consistent with the previous observation that chronic radiation does not increase ROS levels (Fig. S1a), the loss of histones was not affected by inhibiting ROS (Fig. S1b).

### Histone reductions are not abrogated by proteasome or autophagy inhibition

**Figure 5:**
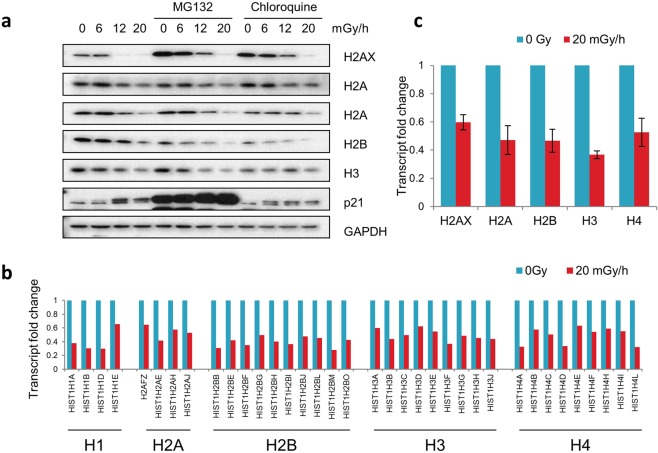
Reduction of histone levels in chronically irradiated cells occurs mainly though reduced transcription, not protein degradation. (**a**) Effect of inhibiting the protein degradation capability in fibroblasts exposed to chronic radiation for 7 days. Proteasome inhibition (5 µM MG132 for 24 h) or lysosomal autophagy inhibition (10 µM chloroquine for 24 h) of fibroblasts exposed to chronic γ-radiation for 7 days. (**b**) Differential expression of histone gene transcripts from RNA-seq analyses of primary fibroblasts after 7 days exposure to 20 mGy/h chronic γ-radiation with changes greater than 1.5-fold and p < 0.01. (**c**) Changes in core histone levels in fibroblasts as measured by qRT-PCR after 7 days exposure to the same radiation conditions as in (**b**).

We next proceeded to investigate how histone levels are reduced in chronically-irradiated cells. As the two major mechanisms of protein degradation in cells occur via the proteasomal or lysosomal (autophagy) pathways, we examined the potential involvement of these by using the chemicals MG132 or chloroquine that inhibit these two pathways, respectively. As seen in Fig. 5a, H2AX protein levels were augmented by these two inhibitors even in un-irradiated cells, indicating the involvement of both the proteasomal and lysosomal pathways in maintaining H2AX levels under steady-state conditions of normal cell growth in culture. Perhaps surprisingly however, this was not the case for other histones, the levels of which were broadly unaffected by MG132 or chloroquine treatment. Regardless of these differences between H2AX and core histones, the levels of H2AX and the other histones declined under chronic radiation conditions, whether cells were grown in the presence of MG132 or chloroquine (Fig. 5a). Collectively, these data indicated that reduction of histone levels under chronic irradiation conditions does not primarily reflect their destruction by proteasomal or lysosomal proteolytic processes. This is a departure from conclusions reached through previous studies on histone loss in senescent cells^57,58^.

### Chronic radiation reduces histone transcript levels

To further explore the mechanism of histone reductions upon chronic irradiation, we considered that this might reflect reduced histone gene transcript levels (via reduced transcription, RNA processing and/or mRNA stability) and ensuing histone protein synthesis. We investigated this by analysing differential gene expression in primary fibroblasts exposed to 6 mGy/h or 20 mGy/h of γ-radiation for 7 days. By RNA sequencing (RNA-seq) analyses, we found irradiation-dose dependent reductions in the levels of all significantly measurable histone transcripts (Fig. 5b). To independently validate these RNA-seq results, we specifically measured levels of selected histone transcripts using qRT-PCR, which confirmed substantial reductions in histone transcripts in response to chronic radiation (Fig. 5c; these data also revealed increased mRNA levels of histone variant H2A.J, whose protein was recently shown to accumulate specifically in senescent human cells^33^, which we also observed in our experiments, Figs. 2c and 3b, although its p-value in the RNA-seq analyses was above that of the set limit so does not appear in final data). These findings thus strongly supported reduced histone gene transcription and/or mRNA stability as being the primary cause of reduced histone levels in chronically-irradiated cells.

### Chronic irradiation leads to chromatin decompaction and elevated global RNA synthesis

**Figure 6:**
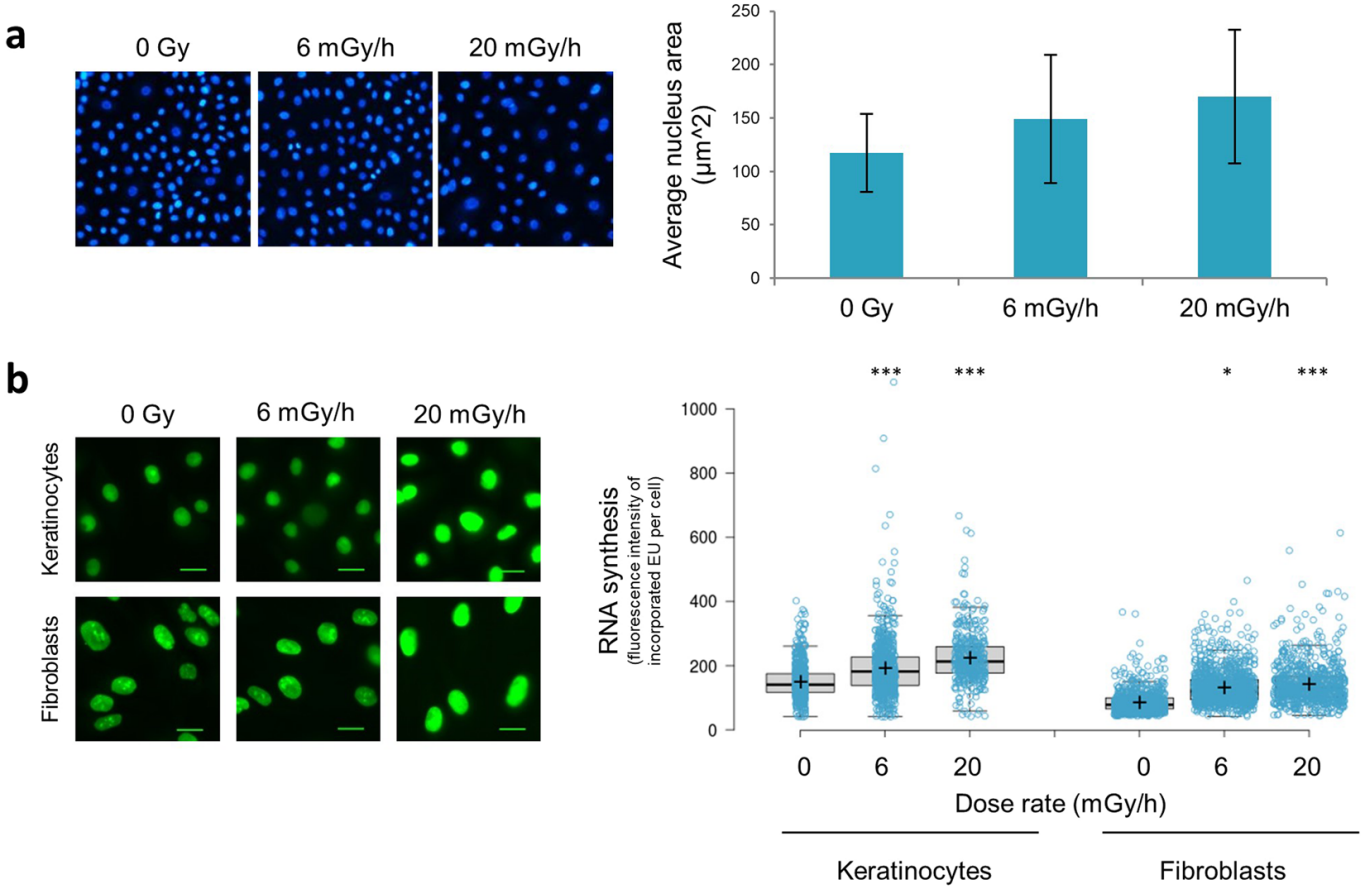
Chronic γ-radiation increases nuclear size and global RNA synthesis. (**a**) Nuclei of fibroblasts exposed to chronic γ-radiation for 7 days, stained with DAPI. All images were acquired by fluorescent microscopy with 10X objective under similar conditions and quantified by automated software detection and nuclear area calculated for >1000 cells. (**b**) Global RNA synthesis in primary keratinocytes and fibroblasts; detected by pulse labelling with Alexa 488-labelled EU and ensuing measurement of average fluorescence intensity per cell. *Is p < 0.1, *** is p < 0.001 relative to un-irradiated samples.

In addition to compacting and packaging of DNA, histones confer higher-order chromatin structure that is pivotal for proper regulation of gene expression. This feature of histones is modulated through modifications such as acetylation and phosphorylation, which in part alter the tightness by which DNA is wound around nucleosomes, and in turn influence access and binding by transcription factors^16,59^. As we found that histone levels are substantially reduced in chronically irradiated cells that have become senescent, we speculated that this might impact on genome compaction and hence transcription. Indeed, when we stained cells with DAPI this revealed that chronic irradiation for 7 days led to a dose-dependent increase in nuclear area (Fig. 6a). As DAPI specifically stains DNA, the increased nuclear area relative to unirradiated cells is indicative of potential increase of nuclear size and therefore looser packaging of chromatin^60^, which would be in keeping with histone reductions. To ascertain the impact of this less compacted DNA on transcription, we measured global RNA synthesis by using the intensity of fluorescent 5-ethynyl uridine (EU) pulse-labelled RNA. This analysis indicated a radiation-dose dependent rise in the amount of overall transcript levels, in both fibroblasts and keratinocytes (Fig. 6b), supporting the notion that reduced histone levels reduce DNA compaction and increase overall RNA synthesis. Clearly, this overall rise in transcript levels does not preclude reduction in the levels of some specific transcripts. Indeed, quantitative RNA sequencing analyses revealed that of the 21 genes whose mRNA levels were most significantly altered by irradiation at 6 mGy/h, 8 of them were up-regulated while the remaining were reduced (Table 3). Chronic irradiation at 20 mGy/h elicited differences in mRNA levels in 304 genes, of which 135 were augmented and the remaining were decreased (Fig. S7).

**Table 3:** RNA-seq differential gene expression.

Down-regulated by 6 mGy/h and 20 mGy/h		Fold-change
EGR1	Early growth response 1	0.266
PTGS2	Prostaglandin-endoperoxide synthase 2	0.394
FCGBP	Fc fragment of IgG binding protein	0.409
IL6	Interleukin 6	0.530
CENPU	Centromere protein U	0.540
CTGF	Connective tissue growth factor	0.541
CYR61	Cysteine rich angiogenic inducer 61	0.561
CCNA2	Cyclin A2	0.584
CENPE	Centromere protein E	0.602
KIF20A	Kinesin family member 20A	0.609
KIF11	Kinesin family member 11	0.617
TOP2A	Topoisomerase (DNA) II alpha	0.622
**Up-regulated by 6 mGy/h and 20 mGy/h**		**Fold-change**
PSG2	Pregnancy specific beta-1-glycoprotein 2	3.436
BTG2	BTG anti-proliferation factor 2	2.378
SPATA18	Spermatogenesis associated 18	2.303
DDIT4	DNA damage inducible transcript 4	2.141
DDB2	Damage specific DNA binding protein 2	1.829
HMOX1	Heme oxygenase 1	1.822
PAPPA	Pappalysin 1	1.792
PPP1R3C	Protein phosphatase 1 regulatory subunit 3C	1.594

List of significant transcript changes (>1.5-fold change, p < 0.01) as identified by RNA-seq analyses of primary fibroblasts chronically irradiated at either 6 or 20 mGy/h for 7 days.

One of the ways that senescent cells influence their environment, and tissue beyond, is through the production and release of proteins related to the senescence-associated secretory phenotype (SASPs) and subsequent inflammatory response^61,62^. At the level of RNA, gene expression associated with SASP (including growth factors FGF2/9, matrix metallopeptidases (MMPs) and collagens) were augmented in chronically-irradiated cells (Table 4). Curiously expression of IL-6, which is a prominent SASP factor, was actually reduced, perhaps reflecting the often-noted lack of consistency between senescent phenotypes and markers^44,63,64^. At the protein level, SILAC identified increases of other SASP proteases and regulators, including metalloproteinases MMP1/2/11/14, TIMP1/2/3, SERPINE1 and Cathepsin B (CSTB), in chronically-irradiated cells (Table 4), indicating the potential involvement of inflammatory processes.

**Table 4:** SASP transcript changes with chronic radiation.

	Fold change 6 mGy/h	Fold change 20 mGy/h	SILAC (protein) or RNA-seq (gene)	Reported direction of change	Reference
IL-6	0.53022	0.4969	RNA only	UP	1
FGF2	—	1.74073	RNA	*Growth factors, UP*	*(1)*
FGF2	0.992	1.153	SILAC	*Growth factors, UP*	*(1)*
FGF9	—	1.54853	RNA	*Growth factors, UP*	*(1)*
IGFBP5	1.317	2.284	SILAC	*IGFBP family, UP*	1
MMP1	3.749	3.147	SILAC	UP	1
MMP2	1.970	1.883	SILAC	*MMP family, UP*	(1), 2
MMP2	—	3.9616	RNA	*MMP family, UP*	(1), 2
MMP3	—	2.00881	RNA	*UP*	1
MMP11	2.462	3.485	SILAC	*MMP family, UP*	(1)
MMP14	1.648	1.550	SILAC	*MMP family, UP*	(1)
TIMP1	1.173	1.337	SILAC	DOWN	1, 2
TIMP2	2.289	1.830	SILAC	UP	1
TIMP3	1.102	1.595	SILAC	*Other TIMPs altered*	(1)
SERPINE1	1.364	1.422	SILAC	UP (PAI-1)	1, 2, 3
CTSB	1.115	1.399	SILAC	UP	1, 2
CTSD	—	1.49723	RNA	Altered	2
FN1	1.383	2.531	SILAC	UP (fibronectin)	1,2
COL2A1	—	13.659	SILAC	*Collagens altered*	*1*
COL13A1	0.653	2.033	SILAC	*Collagens altered*	*1*
COL4A2	1.781	1.339	SILAC	*Collagens altered*	*1*
COL11A1	0.496	1.547	SILAC	*Collagens altered*	*1*
COL8A1	0.850	1.168	SILAC	*Collagens altered*	*1*
COL4A3BP	1.358	1.449	SILAC	*Collagens altered*	*1*
COL6A2	1.120	1.162	SILAC	*Collagens altered*	*1*
COL6A3	0.979	1.004	SILAC	*Collagens altered*	*1*
COL3A1	1.217	1.332	SILAC	*Collagens altered*	*1, 2*
COL1A2	1.239	1.565	SILAC	*Collagens altered*	*1*
COL6A2	—	4.21427	RNA	*Collagens altered*	*1*
COL21A1	—	0.51108	RNA	*Collagens altered*	*1*
COL14A1	—	0.60807	RNA	*Collagens altered*	*1*
COL4A4	—	2.26572	RNA	*Collagens altered*	*1*
LBR	0.760	0.535	SILAC	*Laminins altered*	*1*
TMPO	0.690	0.461	SILAC	*Laminins altered*	*1*
LMNB1	0.789	0.590	SILAC	*Laminins altered*	*1*
TMPO	0.786	0.558	SILAC	*Laminins altered*	*1*
LAMB1	0.916	0.669	SILAC	*Laminins altered*	*1*
LMNB2	0.908	0.767	SILAC	*Laminins altered*	*1*
LAMA2	1.063	0.919	SILAC	*Laminins altered*	*1*
LMNA	1.075	1.040	SILAC	*Laminins altered*	*1*
LAMA5	1.148	0.846	SILAC	*Laminins altered*	*1*
LAMB2	0.969	0.957	SILAC	*Laminins altered*	*1*
LAMA4	0.954	0.954	SILAC	*Laminins altered*	*1*
LMNA	1.063	1.029	SILAC	*Laminins altered*	*1*
LAMA1	1.654	1.198	SILAC	*Laminins altered*	*1*
LAMC1	1.087	1.069	SILAC	*Laminins altered*	*1*

1. Coppe *et al*.^61^; 2. Özcan *et al*.^81^; 3. Vaughan *et al*.^82^; (pathway, not direction).

## Discussion

Although many aspects of cellular responses to radiation-induced DNA damage have been well-characterised, there remain areas such as chronic radiation effects on cells, that are yet to be fully explored and understood. This report demonstrates that the current understanding of cellular responses to IR, gained from studies of acute radiation, would not have predicted the effect of chronic radiation on very fundamental cellular characteristics such as cell proliferation and senescence. According to the standard model of cellular responses to radiation, DNA breaks – and in some cases even a single DSB – would trigger a temporary cell cycle arrest, allowing repair before continued progression through the cell cycle^65–67^. While this may understandably retard cell division to some extent, we did not expect it to trigger substantial cellular senescence because in our experiments, IR was delivered at doses that are estimated to inflict fewer than a single DSB per cell each hour. This very low and infrequent estimated number of DSBs was confirmed using 53BP1 foci as a surrogate measurement of DSB formation in chronically-irradiated cells in culture. Although acute radiation-induced cellular senescence has been previously observed and documented^18^, it was assumed to occur when cells fail to repair their damaged DNA. This can happen if the DNA damage is too complex for straightforward repair or if the breaks are too numerous for the cells to cope. In view of low radiation doses such as we used in our studies, the latter is unlikely. Regarding the former, IR can indeed cause clustered DNA damage that can be a mixture of DSBs, single-strand breaks and base damage^68,69^, which can be difficult to repair. If this were the case in our studies however, the number of DSB foci would be expected to accumulate beyond the expected steady-state number, which was not the case. Our findings with chronic low-dose irradiation bring into focus the potential clinical effects of chronic low dose radiation, especially regarding pathologies that are promoted by or exacerbated by senescent cells. Although senescence has been identified in cellular models as a result of chronic radiation^70–72^, any potential effects in humans or animal models may have previously escaped notice as senescent cells accumulate naturally in the body with age^52,73^. Hence any additional contribution of chronic low dose radiation may not be immediately obvious and appreciated.

A key finding from our studies is that the amounts of H2AX and core histones are reduced in chronically-irradiated cells. Histone loss in yeast cells treated with zeocin or acute high dose radiation was reported by Hauer *et al*.^57^. They demonstrated this to be due to proteolytic degradation of the histone proteins; which is also the route by which histone levels were reportedly reduced in Jun-deficient mouse fibroblasts that were exposed to high levels of reactive oxygen species^74^. Our findings suggest that chronically-irradiated cells employ a different means to the same end: namely by reducing histone gene mRNA levels. There are various factors that could explain this apparent difference in regulatory mechanism between the various studies, ranging from species used, genetic modification, the nature of DNA damaging agent and/or dosing schedule; none of which can be singled out without empirical testing. A feature deserving more attention in future studies will be to establish precisely how histone gene transcripts become reduced in response to chronic radiation, for example via decreased transcription, reduced transcript stability, or both. Since most histone gene expression is usually up-regulated during S-phase^75,76^, it is likely that a contributing factor to histone transcript reductions we observe upon chronic irradiation could reflect chronically irradiated cells having lower S-phase indices because they have arrested cell cycle or entered senescence. However, the promoter of H2AX is not cell cycle-responsive^76^ and yet Fig. 5 shows its transcript levels declined in concert with other histones in response to chronic radiation. We also demonstrate the consistent loss of H2AX protein, which does not fluctuate with cell cycle but has been shown to be stabilised by DNA-damage^77^. Identifying how the multiple histone transcript levels are co-ordinately regulated in such settings could potentially uncover a master regulator(s) that actively suppresses histone gene expression in senescent cells.

Independent of the mechanism, reduction of histone levels has a clear predicted outcome – increased relaxation of chromatin and a concomitant general increase in transcription, both of which we indeed detected in chronically-irradiated cell populations (Fig. 6). It is now established that senescent cells are not benign and, in many circumstances, are actively destructive^78,79^. This is in part connected to them producing and secreting proteins, especially pro-inflammatory ones that can cause the dysfunction of surrounding cells and tissues, plus those further away if secreted into the bloodstream^62^. We have indeed detected chronic radiation-induced increased transcripts for some SASPs as listed in Table 4, although we have also noted a reduction of IL-6 transcripts. This is, however, consistent because IL-6 is not part of the Skin Ageing-Associated Secreted Proteins (SAASP)^80^ which, as the name suggests, are proteins secreted by ageing human dermal fibroblasts, the very cells used here. In summary, our results reveal histone reductions associated with cellular senescence as an effect of continuous exposure to low doses of ionising radiation that may have wider implications for ageing and the incidence of specific pathologies.

## Supplementary information

Supplementary figures.

SILAC data.

RNA-seq differential gene expression.

RNA-seq counts.
