# Supplementary material for: Chronic irradiation of human cells reduces histone levels and deregulates gene expression
Source: Sci Rep. 2020 Feb 10;10:2200. doi: 10.1038/s41598-020-59163-4 (PMC7010678; doi:10.1038/s41598-020-59163-4)
Supplement: Supplementary file 1 — Supplementary figures. [file 41598_2020_59163_MOESM1_ESM.pdf]

Supplementary Information

Chronic irradiation of human cells reduces histone levels and deregulates gene expression

Donna J. Lowe*[1, 2], Mareike Herzog[2], Thorsten Mosler[3], Howard Cohen[4], Sarah Felton[5], Petra Beli[3], Ken Raj[1], Yaron Galanty*[2] and Stephen P. Jackson*[2]

[1] Radiation Effects Department, Centre for Radiation, Chemical and Environmental Hazards, Public Health England, Chilton, Didcot, Oxfordshire, OX11 0RQ, UK

[2] Wellcome/Cancer Research UK Gurdon Institute and Department of Biochemistry, University of Cambridge, Cambridge, CB2 1QN, UK

[3] Institute of Molecular Biology (IMB), 55128 Mainz, Germany

[4] Elizabeth House Surgery, Warlingham, Surrey, CR6 9LF, UK

[5] Department of Dermatology, Churchill Hospital, Oxford, OX3 7LJ, UK

*Corresponding author

Email: Donna.Lowe@phe.gov.uk; s.jackson@gurdon.cam.ac.uk; y.galanty@gurdon.cam.ac.uk

a

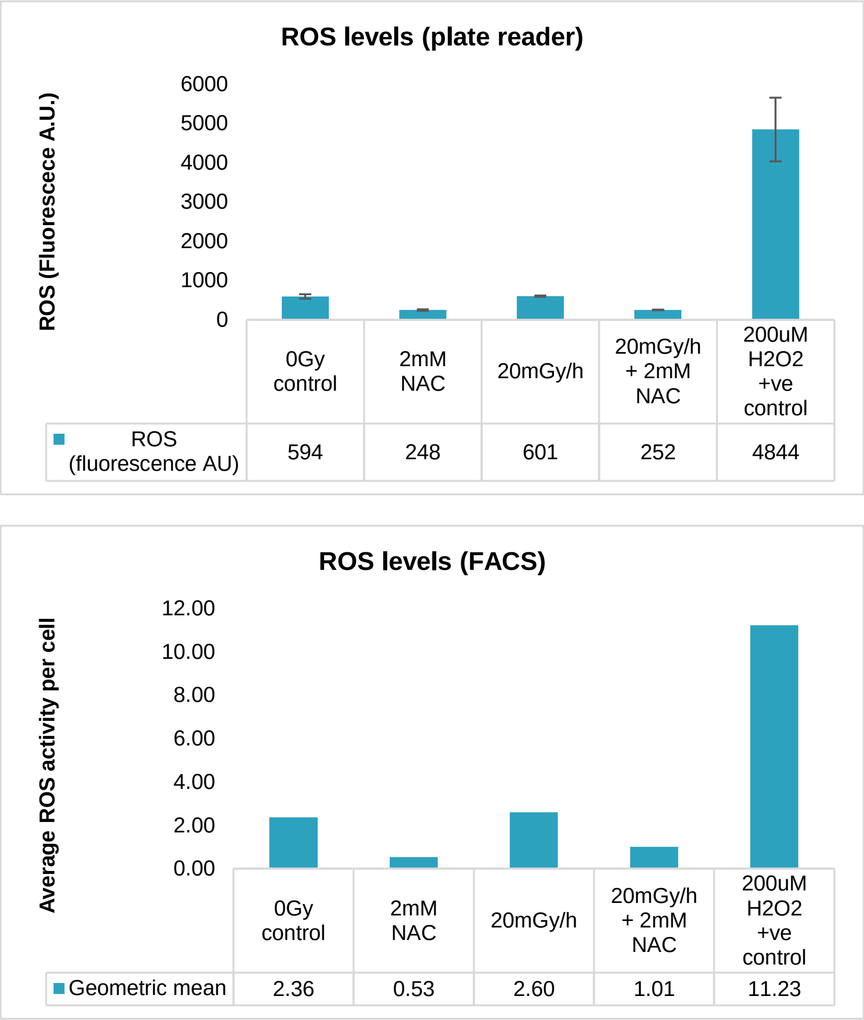

b

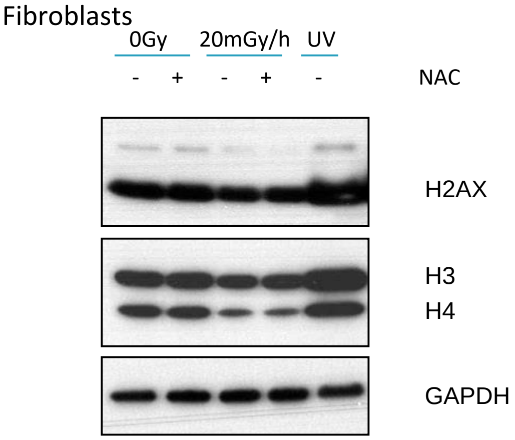

Supplementary figure 1. Reactive oxygen species levels are not increased in chronically irradiated cells

a, ROS levels in primary fibroblasts exposed to chronic radiation at 20 mGy/h for 7 days, with/without N-acetyl-Cysteine ROS inhibitor, plus hydrogen peroxide positive control. Measured by plate reader of cell population (upper) or flow cytometry of individual cells (lower). **b,** histone levels in the same cells as ‘a’.

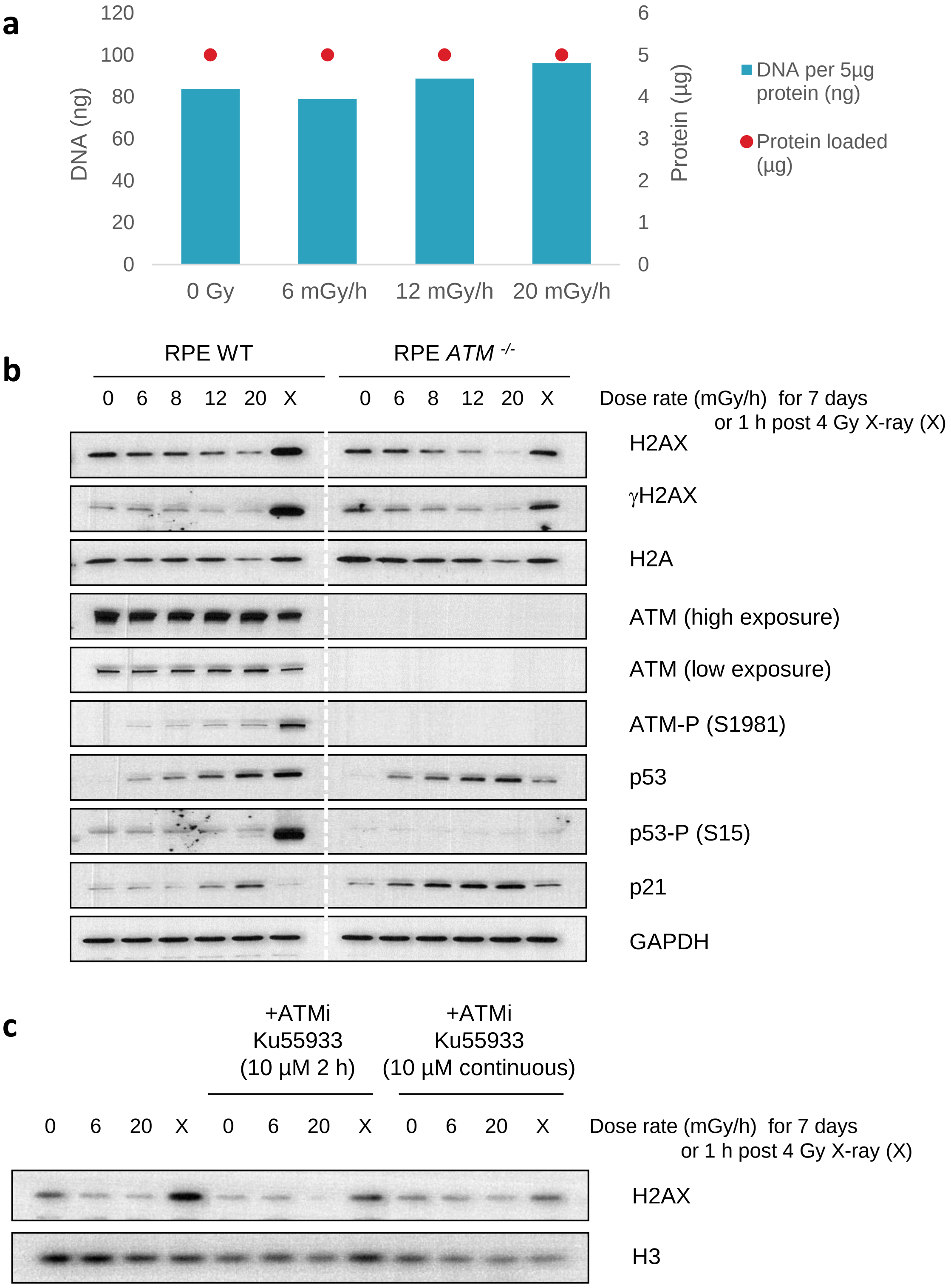

Supplementary figure 2. Chronic radiation-induced histone reductions do not depend on ATM activity

a, Amounts of cellular double-stranded DNA per 5ug of total protein from each cell lysate, measured using SYBR Gold fluorescence dye. **b,** Chronic radiation-induced changes in wild-type or *ATM* knockout RPE-1 cells, exposed to 20 mGy/h chronic radiation at stated dose rates for 7 days. **c,** Inhibition of ATM with 10 µM Ku55933 in primary fibroblasts chronically irradiated for 7 days, treated either for 2 h prior to harvesting or for the duration of the 7-day chronic radiation exposure.

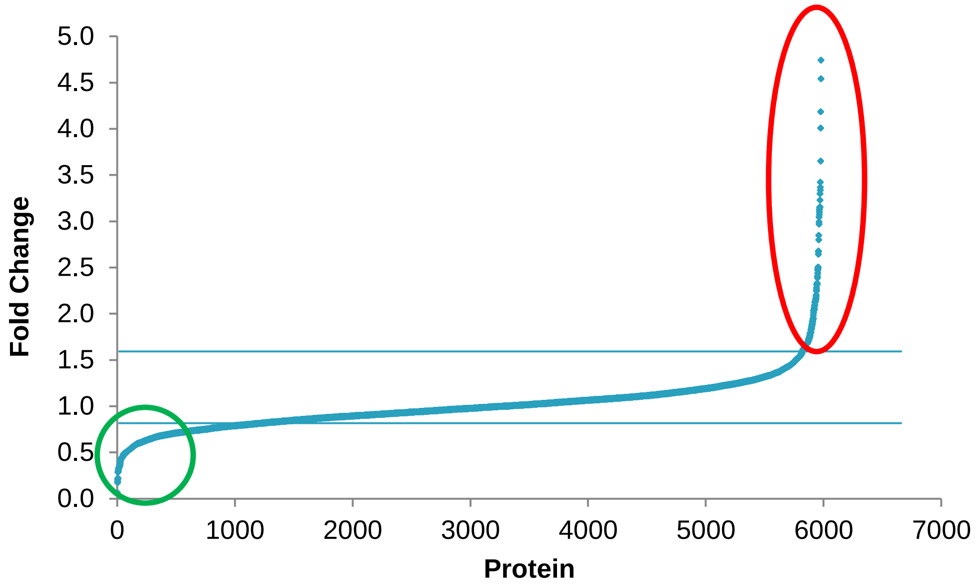

Downregulated

COL12A1
HIST1H1A
NCAPG
HIST1H1D
WDHD1
HMGB2
CEBPD
LBR
RIF1
CADM1
SVEP1
NCAPD2
GABPA
TMPO
SMC2
ATAD3B
SPC24
MCM3
C7orf50
SMC4
SSNA1
H2AFV;H2AFZ
MCM5
NCOA4
HIST2H2AB
PARP1
HIST1H1C
MCM4
PHF2
MCM6
TOP2A
SMC6
MCM7

MCM2
HIST1H1B
LUC7L
C19orf53
SPC25
PSMB9
CHD1L
PRC1
RBPJ
PSIP1
DNMT1
DDX39A
LMNB1
TRIP13
PLIN2
HIST1H4A
HMGB3
MDC1
HMGB1
HIST2H3A;HIST1H3A;HIST3H3
HIST2H2BE;HIST1H2BB;HIST1H2BO
AAAS
RFC2
DNAJC9
IFITM2
SCAF4
HIST1H2BC;HIST1H2BN;HIST1H2BM;HIST1H2BH;HIST2H2BF;HIST1H2BD;HIST1H2BL

CDK2
HIST1H1E
H2BFS
COLEC12
PAK1IP1
POLD1
HIST2H2AC;HIST2H2AA3;HIST1H2AJ;HIST1H2AH;H2AFJ;HIST1H2AG;HIST1H2AD
STMN1
RFC3
CDK1;CDC2
DEK
TOP1
TMPO
SYNE2
BRD1
RBM42
MAD2L1
HNRNPH3
EIF4A2
TWSG1
SMARCAD1
H1FX
MSH6
H2AFX
CHD8
BRD4
DDX50
FEN1
RCOR1

UBA7
CDC73
CCDC137
NOLC1
CPNE2
HMGN2
SHMT2
CRELD2
POLDIP3
RPL22L1
KDM1B
TEAD3
NUMA1
RFC4
CAND2
RPRD1B
HNRNPC
ARID1A
CBX5
MED17
GNE
IRF2BP2
PDS5B
SUGP2
MPG
PTMA
TRPT1
FAM50A
BAZ1B
SIN3A
SUPT16H
SMC3
GLYR1

MDN1
UBTF
RECK
DCPS
CTCF
LEPREL4
MED12;TNRC11
PRPF4

Upregulated

CALB2
FN1
HSPB7
SERPINB2
IGFBP5
AKR1C2
PERP
WDR13
AKR1C1
ADIRF
RBM3
SNTA1
EPS8L2
NAGLU
DES
SNCG
PGPEP1
XRCC4
RBBP9
GSTM4;GSTM1
CEP170B
RND3
AKR1D1
SVIL
SSH3
AK1
TBC1D2
RRM2B
HERC4
GLRX
FBXO44
UCHL1

Histones
Replication/mitosis
Chromatin associated
Nuclear/DNA regulation

Structural/Metabolism
Ubiquitin/proteasome
Caner/anti-proliferation
DDR/apoptosis/arrest

Supplementary figure 3. SILAC protein changes following 20 mGy/h chronic IR for 7 days
Significant and consistent changes determined by proteins with >1.5 mean fold change and the same direction of change in all triplicate samples; assigned to main function and ordered by fold-change.

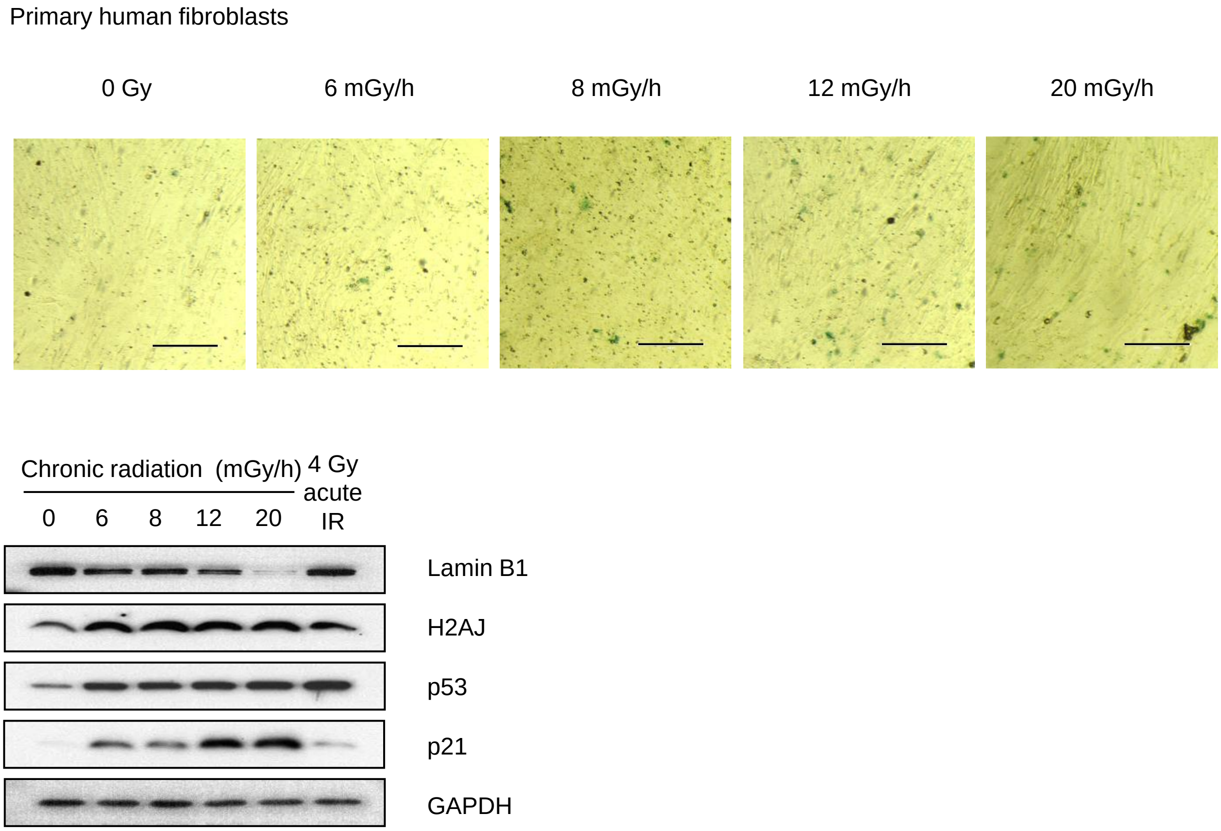

Supplementary figure 4. Chronic radiation-induced senescence
β-galactosidase staining of primary fibroblasts exposed to chronic radiation at stated dose rates for 7 days (upper panel) and immunoblotting of protein extracts of these samples (lower panel).

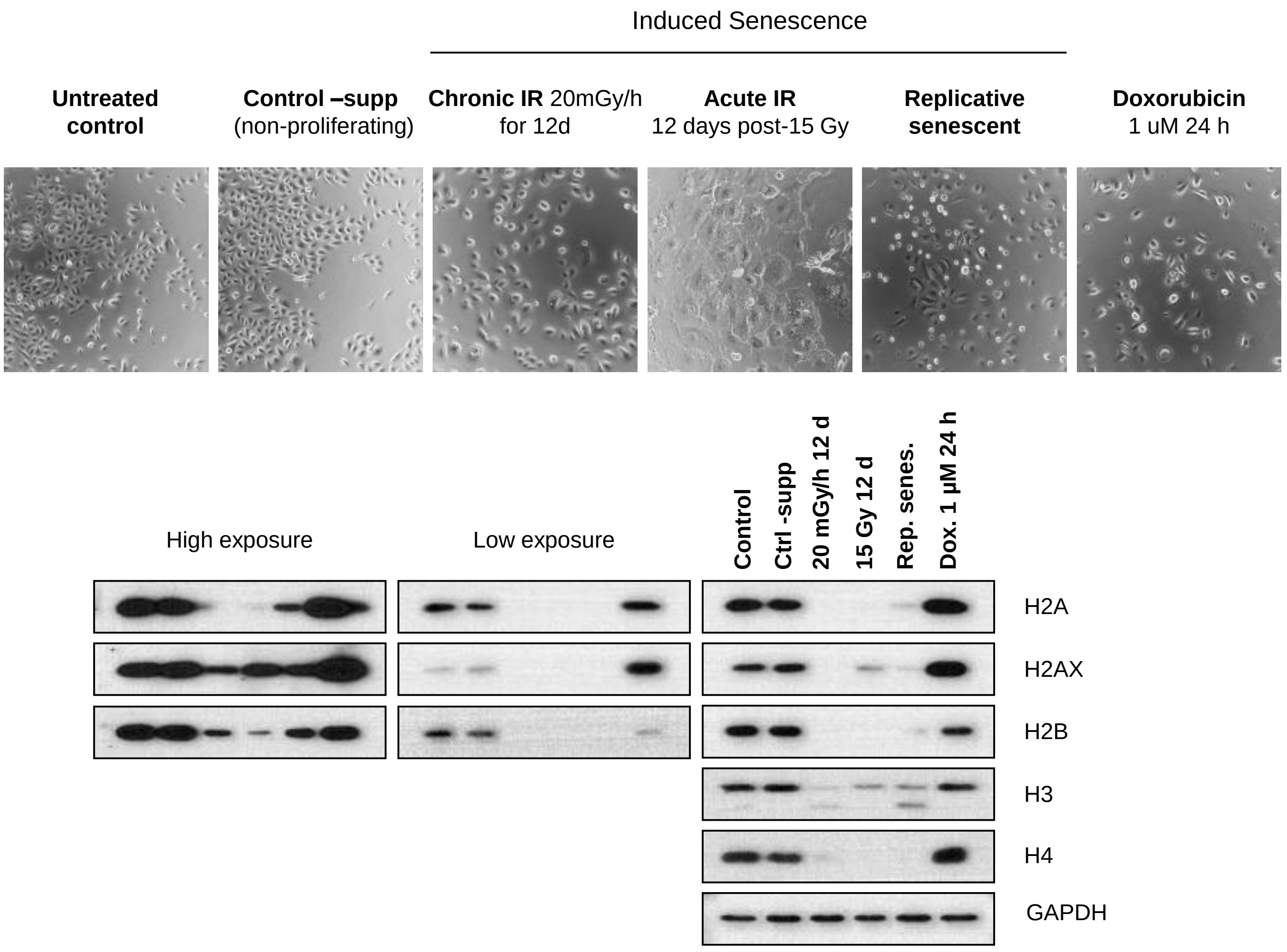

Supplementary figure 5. Histone changes in senescent keratinocytes
Primary keratinocytes cultured in sub-confluent conditions. Untreated cells, either proliferating or not (via withdrawal of culture media supplements), and senescent cells induced by exposed to chronic or acute radiation, or passaged until replicative senescence reached. Phase contrast images of cells at harvesting (upper panel) were acquired using EVOS microscope with 10X objective and western blot analysis of histone levels in these cells (lower panel).

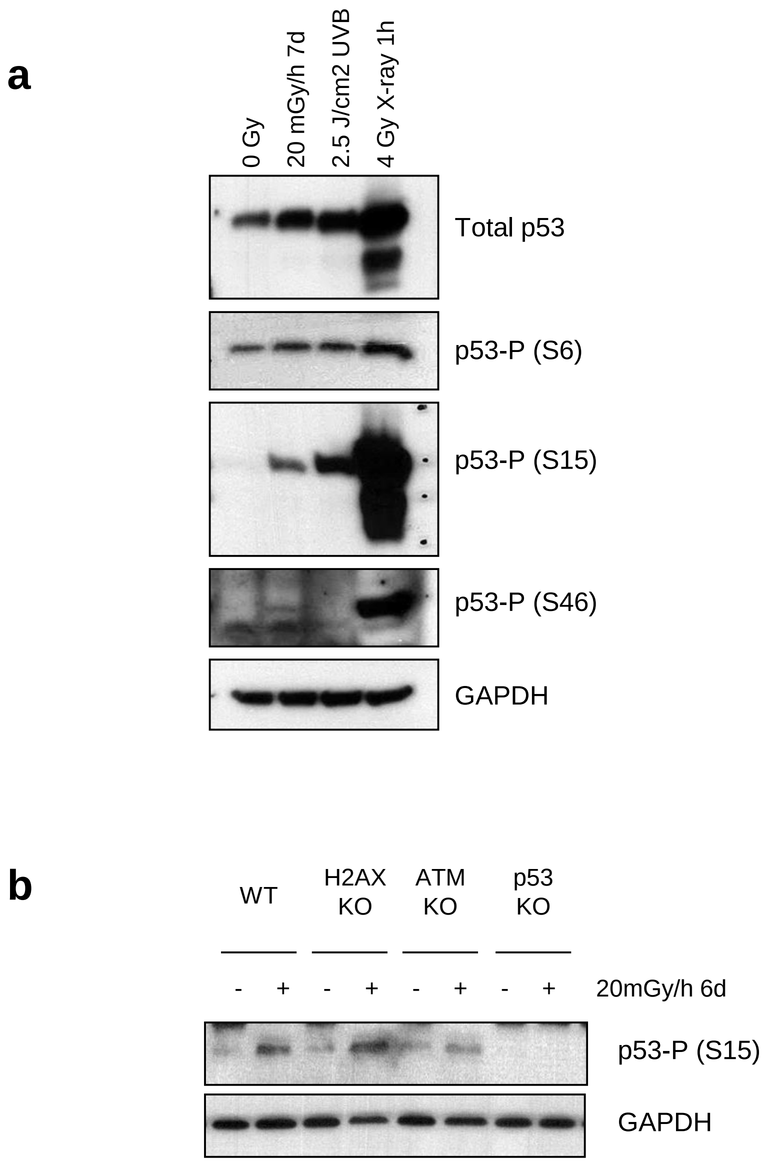

Supplementary figure 6. p53 phosphorylation in chronically irradiated cells
a, Total p53 and phosphorylation of p53 in response to chronic radiation at 20 mGy/h for 7 days, UVB irradiation (2.5 J/cm^2, 312nm). Lanes 1-3 fibroblasts, lane 4 RPE-1 cells. **b,** phosphorylated p53 at serine 15 in chronically irradiated RPE cells.

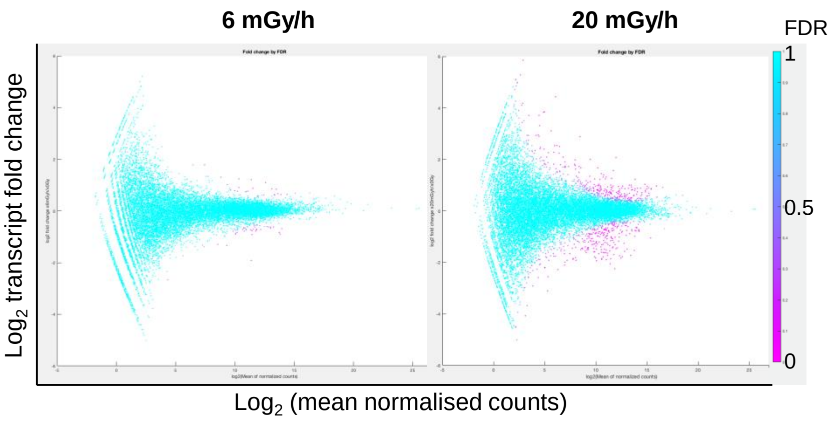

21 Genes

Downregulated = 13
Upregulated = 8

304 Genes

Downregulated = 169
Upregulated = 135

Supplementary figure 7. RNA-seq data analysis
Significant differential gene expression changes from all donors per sample type with adjusted p-value (FDR) <0.01; fold change >1.5
